# AttABseq: an attention-based deep learning prediction method for antigen–antibody binding affinity changes based on protein sequences

**DOI:** 10.1093/bib/bbae304

**Published:** 2024-07-03

**Authors:** Ruofan Jin, Qing Ye, Jike Wang, Zheng Cao, Dejun Jiang, Tianyue Wang, Yu Kang, Wanting Xu, Chang-Yu Hsieh, Tingjun Hou

**Affiliations:** College of Pharmaceutical Science, Innovation Institute for Artificial Intelligence in Medicine of Zhejiang University, Zhejiang University, Yuhangtang Road 866, Hangzhou 310058, Zhejiang, China; College of Life Science, Zhejiang University, Yuhangtang Road 866, Hangzhou 310058, Zhejiang, China; College of Pharmaceutical Science, Innovation Institute for Artificial Intelligence in Medicine of Zhejiang University, Zhejiang University, Yuhangtang Road 866, Hangzhou 310058, Zhejiang, China; College of Pharmaceutical Science, Innovation Institute for Artificial Intelligence in Medicine of Zhejiang University, Zhejiang University, Yuhangtang Road 866, Hangzhou 310058, Zhejiang, China; College of Computer Science and Technology, Zhejiang University, Yuhangtang Road 866, Hangzhou 310058, Zhejiang, China; College of Pharmaceutical Science, Innovation Institute for Artificial Intelligence in Medicine of Zhejiang University, Zhejiang University, Yuhangtang Road 866, Hangzhou 310058, Zhejiang, China; College of Pharmaceutical Science, Innovation Institute for Artificial Intelligence in Medicine of Zhejiang University, Zhejiang University, Yuhangtang Road 866, Hangzhou 310058, Zhejiang, China; College of Pharmaceutical Science, Innovation Institute for Artificial Intelligence in Medicine of Zhejiang University, Zhejiang University, Yuhangtang Road 866, Hangzhou 310058, Zhejiang, China; College of Pharmaceutical Science, Innovation Institute for Artificial Intelligence in Medicine of Zhejiang University, Zhejiang University, Yuhangtang Road 866, Hangzhou 310058, Zhejiang, China; College of Pharmaceutical Science, Innovation Institute for Artificial Intelligence in Medicine of Zhejiang University, Zhejiang University, Yuhangtang Road 866, Hangzhou 310058, Zhejiang, China; College of Pharmaceutical Science, Innovation Institute for Artificial Intelligence in Medicine of Zhejiang University, Zhejiang University, Yuhangtang Road 866, Hangzhou 310058, Zhejiang, China

**Keywords:** antigen–antibody binding affinity change, therapeutic antibody, antibody optimization, artificial intelligence, deep learning

## Abstract

The optimization of therapeutic antibodies through traditional techniques, such as candidate screening via hybridoma or phage display, is resource-intensive and time-consuming. In recent years, computational and artificial intelligence-based methods have been actively developed to accelerate and improve the development of therapeutic antibodies. In this study, we developed an end-to-end sequence-based deep learning model, termed AttABseq, for the predictions of the antigen–antibody binding affinity changes connected with antibody mutations. AttABseq is a highly efficient and generic attention-based model by utilizing diverse antigen–antibody complex sequences as the input to predict the binding affinity changes of residue mutations. The assessment on the three benchmark datasets illustrates that AttABseq is 120% more accurate than other sequence-based models in terms of the Pearson correlation coefficient between the predicted and experimental binding affinity changes. Moreover, AttABseq also either outperforms or competes favorably with the structure-based approaches. Furthermore, AttABseq consistently demonstrates robust predictive capabilities across a diverse array of conditions, underscoring its remarkable capacity for generalization across a wide spectrum of antigen-antibody complexes. It imposes no constraints on the quantity of altered residues, rendering it particularly applicable in scenarios where crystallographic structures remain unavailable. The attention-based interpretability analysis indicates that the causal effects of point mutations on antibody–antigen binding affinity changes can be visualized at the residue level, which might assist automated antibody sequence optimization. We believe that AttABseq provides a fiercely competitive answer to therapeutic antibody optimization.

## Introduction

Antibody-based therapy is a highly effective approach for treating various diseases, such as cancer and infections by pathogens [1–3]. In recent years, certain forms of immunotherapies, including convalescent plasma and monoclonal antibodies, have been identified as effective therapeutic options for the treatment of coronavirus disease 2019 (COVID-19) [4–6]. In comparison to alternative therapeutic approaches, antibody therapies possess notable benefits, such as high specificity, low toxicity, and strong efficacy [7, 8]. Despite these great merits, it is important to note that the majority of these antibodies are not naturally occurring and therefore require meticulous screening, design, or optimization within a laboratory setting [9–11]. The optimization of therapeutic antibodies by conventional experiments is time-consuming and laborious [12, 13]. For example, low-throughput screening of full-length antibodies based on mammalian systems expressed in cells usually results in a problem of affinity ceiling of an antibody [14], while *in vitro* methods for affinity maturation are more sensible but inefficient [12, 15].

The process of antibody optimization encounters challenges due to the massive diversity space of antibody sequences, which can lead to a combinatorial explosion of potential mutations. This complexity is further compounded when dealing with multi-chains and multi-points maturation, making it challenging to experimentally test a huge number of antibody variants and identify optimized leads [16, 17]. To prevent excessive resource consumption and accelerate the pace of development, it is imperative to construct computational models capable of efficiently identifying antibody variants with superior binding affinity to a certain antigen. The utilization of machine learning (ML) technologies has shown great potential in antibody engineering [18–21].

Antibody optimization poses an intricate scientific endeavor that necessitates a comprehensive multi-objective approach, taking into account crucial factors such as solubility, stability, and specificity. The success of this optimization relies heavily on deciphering the alterations in antibody affinity changes due to point mutation (ΔΔG) induced by missense mutations. Presently, methods for predicting ∆∆G upon mutations can be broadly categorized into two classes: force field–based and ML-based. FoldX [22] is a sophisticated computational software utilized for protein structure and stability calculations, has been considered one of the most popular force field–based methods and has frequently been used as a benchmark. The primary function of FoldX is the accurate prediction of binding free energy changes in protein–protein and protein–ligand interactions. BeAtMuSiC [23], an ML–based tool, uses a statistical potential extrapolated from established protein structures to estimate the impact of mutations on both the interaction potency at the interface and the global stability of molecular complex [23]. Both of these tools rely on physical and statistical models for predictions related to protein structure and stability, which may introduce certain limitations in prediction accuracy. A variety of methods based on ML and deep learning (DL) techniques have been developed, such as mCSM-AB [24], TopNetTree [25], PerSpect_EL [26], and GeoPPI [27], and they exhibited improved performance compared with force field–based methods. mCSM-AB is an ML model that specifically predicts antibody affinity changes with graph-based signatures of antigen–antibody complexes, and it also offers a user-friendly web server for in silico antibody optimization. TopNetTree and PerSpect_EL incorporate element- and site-specific persistent homology techniques to address the intricate structural complexity within protein–protein complexes. These techniques provide critical biological insights within topological invariants, utilizing ML and DL to embed protein topological features and achieving high accuracy in predicting affinity changes. GeoPPI is an antibody affinity changes predictor based on a pre-trained graph neural network, capable of learning important features characterizing interactions between atoms in protein structures. Although the aforementioned models provide solutions for various antibody optimization challenges, their reliance on high-quality crystal structure data from antigen–antibody complexes limits their applicability in situations where only sequence information is accessible.

Recently, there has been growing interest in the application of cutting-edge DL algorithms, such as Transformer-based BERT [28] and GPT [29], in diverse fields ranging from natural language processing to bioinformatics and computational biology [30–35]. Previous studies have shown that large-scale pre-trained protein language models, such as ESM [36] and TAPE [37], have the ability to accurately predict various properties of different proteins solely based on their FASTA sequences, including three dimensional (3D)-folded structures [38, 39], hydrophobicity, and other physical and biochemical properties [40]. Furthermore, efforts are underway to expand the utility of DL models in predicting protein–protein interactions and determining the 3D structures of protein complexes [41–44]. The advancements in these studies foster optimism regarding the potential of sequence-based antibody optimization.

However, predicting the intricate structural modifications and ∆∆G changes arising from mutations in an antigen–antibody complex presents substantial challenges, primarily owing to the exceptional adaptability of loops within the complementarity-determining regions (CDRs) of antibodies that interact primarily with the antigen [45, 46]. In fact, accurately determining the biochemical properties and structural conformation of antibodies and antigen–antibody complexes remains highly challenging [47–49] despite the employment of advanced techniques like Alphafold2 [50] and Alphafold-multimer [51]. The pressing need is for the advancement of cutting-edge DL-based methods specifically designed for antibody optimization, thereby enhancing the competitiveness of our approaches.

In this study, we propose AttABseq, an attention-based DL method for optimizing a wide range of antibodies solely using sequence information from antigen–antibody protein complexes, offering a fast and convenient way for antibody optimization without requiring structural data. It is a pioneering DL method only based on full-length sequences capable of optimizing diverse antibody types against multiple antigens, relying on publicly available antigen-antibody complex data. AttABseq comprises three fundamental components: the embedding block, the attention block, and the predicting block. These elements handle distinct tasks: the embedding block focuses on protein sequence feature encoding, the attention block integrates crucial information from protein complexes, and the predicting block forecasts affinity changes. This enables the accurate computation of antigen–antibody binding affinity variations solely based on amino acid mutations and protein complex sequences. The attention block, drawing from protein-folding principles, is particularly critical in refining affinity change predictions by emphasizing intricate interaction details and amino acid modifications within a single protein complex. AttABseq achieves state-of-the-art results compared with existing sequence-based models in K-fold cross-validation. Even as data heterogeneity increases because of a label-ascending ordered split testing methodology, it continues to demonstrate excellent affinity prediction ability, surpassing widely used force field–based methods like FoldX [22]. AttABseq also allows us to comprehend the impact of point mutations on antigen–antibody affinity changes at the residue level, potentially aiding in automated antibody sequence optimization. In summary, AttABseq offers a sequence-centric approach for optimizing various antibody type and displays exceptional generalization capabilities.

## Results

The K-fold cross-validation and label-ascending partition validation of the AttABseq’s performance were conducted on three datasets: two containing single point mutants and the other one containing multiple point mutants. Here, we use the Pearson correlation coefficient (PCC) and R-squared (R^2^) to evaluate the model’s prediction ability (see Methods for details). Our model consistently outperformed structure–based and sequence-based methods across all three datasets. Furthermore, the results of a uniform feature transformation approach (see Methods for details) indicated that the attention block of AttABseq displayed a significant propensity for assigning weights to mutated residues. This evidence unveiled the ability of AttABseq to effectively capture the impact of individual residues on affinity alterations, thereby enhancing its reliability and utility in predicting molecular interactions.

### The architecture of AttABseq

Attention-based networks have demonstrated significant effectiveness in capturing the intricate interactions among entities [52–55]. Therefore, these networks are particularly well-suited for modeling proteins, as the functionality of proteins is primarily influenced by the interactions among their constituent residues [56, 57]. To enhance our ability to model interactions between antigens and antibodies, we have developed AttABseq, a DL model comprising three key components: an embedding block, an attention block, and a predicting block. Each component plays a distinct role, collectively enhancing the overall efficacy of AttABseq. Detailed information about each block is provided in the Methods section.

**Figure 1:**
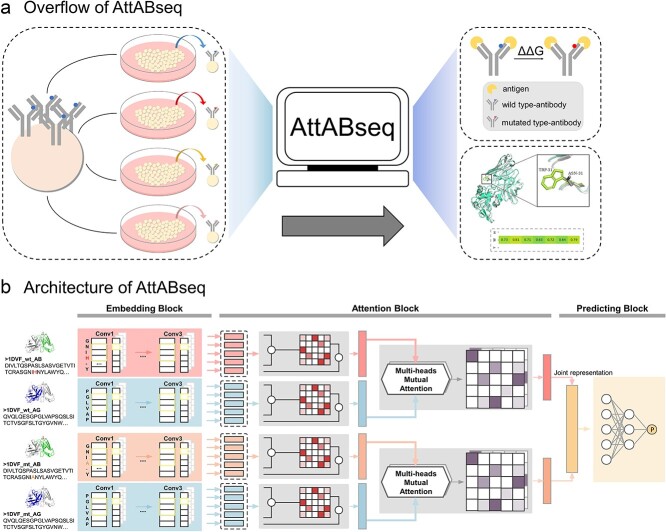
(a) Overflow of AttABseq. Antibody data, encompassing various sequence compositions resulting from mutations, are fed into AttABseq; the model efficiently computes affinity change values based on amino acid mutations and offers pertinent inferences regarding residue interaction that underlies the shifts in antigen–antibody affinity; (b) Architecture of AttABseq; AttABseq is structured into three key components, ordered from left to right: the embedding block, the attention block, and the predicting block. The embedding block comprises three distinct convolutional neural networks, each employing a unique kernel size. The attention block, on the other hand, is comprised of two essential components: self-attention and multi-head mutual attention. Finally, the predicting block seamlessly amalgamates data from both the wild-type and mutated-type protein complexes, culminating in the ultimate prediction of ΔΔG.

Figure 1a illustrates the core objective of the model. The model efficiently processes input data, consisting of the sequence information for both the antigen–antibody complex and the details on amino acid mutations. It then delivers precise predictions of binding affinity changes associated with the mutated residues. Additionally, it provides a logically reasoned inference diagram of antigen–antibody affinity changes. Figure 1b presents a comprehensive representation of the AttABseq model’s architectural components. It initiates with the acquisition of sequence data for both the antigen and antibody proteins within the wild-type and mutated antigen–antibody complexes. Subsequently, it calculates the one-hot matrix and position-specific scoring matrix (PSSM) for each protein. These computed features are thoughtfully concatenated as the input for the AttABseq model. This sophisticated model is then employed to predict the affinity change for protein complexes, encompassing both the wild-type and mutated variants. The embedding block of the model employs three convolutional neural networks, each with varying kernel sizes, to systematically extract the essential sequence-based features. The attention block meticulously examines the interplay between antigen and antibody sequences, aiming to capture valuable interaction data related to the protein complex. Lastly, the predicting block integrates the aforementioned information, culminating in the estimation of ∆∆G as a consequence of residue mutations.

### Performance comparisons on datasets with mutations in K-fold cross-validation

**Figure 2:**
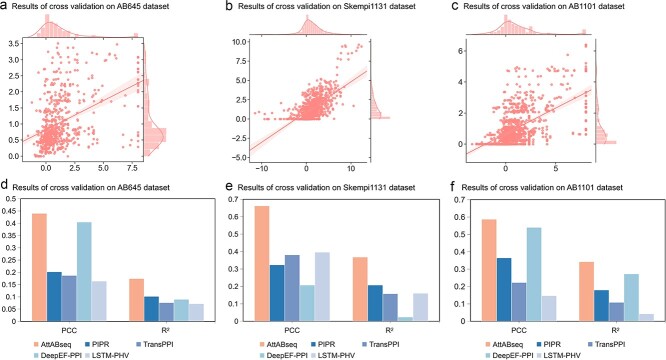
Performance comparison in the K-fold cross-validation experiment. The evaluation metrics are PCC and R^2^; (a) Scatterplot of the ten-fold cross-validation performance on AB645; (b) Scatterplot of the ten-fold cross-validation performance on S1131; (c) Scatterplot of the five-fold cross-validation performance on AB1101; (d) Comparison of the ten-fold cross-validation performance with four sequence-based methods on AB645; (e) Comparison of the ten-fold cross-validation performance with four sequence-based methods on S1131; (f) Comparison of the five-fold cross-validation performance with four sequence-based methods on AB1101.

Cross-validation was used to train the AttABseq model and other sequence models based on the same datasets. This approach facilitates the objective evaluation and comparative analysis of AttABseq’s performance. Given the absence of an algorithm that exclusively leverages protein sequence data across a wide spectrum of antigen–antibody complexes, our study undertakes a comparative assessment between AttABseq and well-established protein interaction models, namely PIPR [58], TransPPI [59], DeepFE-PPI [60], and LSTM-PHV [61]. Notably, all of these models solely rely on protein sequence data as their input and have publicly available source codes. Our model’s performance on the AB645 dataset used by AB-Bind [62] yielded a PCC of 0.440, surpassing DeepFE-PPI by a notable 8.7% in five-fold cross-validation, as shown in Fig. 2a and d. Furthermore, on the S1131 dataset from Skempi 2.0 [63], our model achieved a PCC of 0.663 as shown in Fig. 2b and e. In a ten-fold cross-validation setting, this performance exceeded that of LSTM-PHV by an impressive 67.1%, as illustrated in Fig. 2d and e. Additionally, when evaluated on the AB645 dataset using the five-fold cross-validation, the AttABseq model exhibited a R^2^ value of 0.174, which surpasses the performance of PIPR by a substantial margin of 70.7%. Similarly, on the S1131 dataset with the 10-fold cross-validation, the AttABseq model achieved an R^2^ value of 0.368, outperforming PIPR by an impressive 77.2%. These results unequivocally demonstrate the superior accuracy of AttABseq in predicting ∆∆G alterations resulting from amino acid mutations when compared to alternative models.

To comprehensively assess the performance of protein sequences exhibiting varying numbers of amino acid mutations, we incorporated the AB1101 dataset as described in the Methods section. This dataset comprises 645 single-point mutations and 456 multi-point mutations across 32 distinct antigen–antibody complexes. Notably, the model we have developed consistently outperforms the other sequence-based models, as exemplified in Fig. 2c and f. The performance of AttABseq on the AB1101 dataset achieves a PCC of 0.587, marking an 8.7% enhancement over the performance of DeepFE-PPI. This underscores AttABseq’s capacity to maintain prediction stability in datasets characterized by a myriad of point mutations, an attribute attributed to the attention block’s proficiency in capturing antigen–antibody interaction alterations due to amino acid substitutions. This stability indeed bodes well for the advancement of sophisticated antibody optimization methodologies.

### Performance comparisons on datasets with label-ascending ordered split

Collis et al. [64] have highlighted the notable disparities in sequence information within antigen and antibody complexes in authentic biological contexts. This observation underscores the higher degree of data variation inherent in real-world datasets. Furthermore, variations in antibody residue mutations yield distinct alterations in energy levels. To achieve practical utility in real-world scenarios, a DL model must demonstrate robust generalization capability. Therefore, it is essential to assess the model’s response to variations in data heterogeneity.

**Figure 3:**
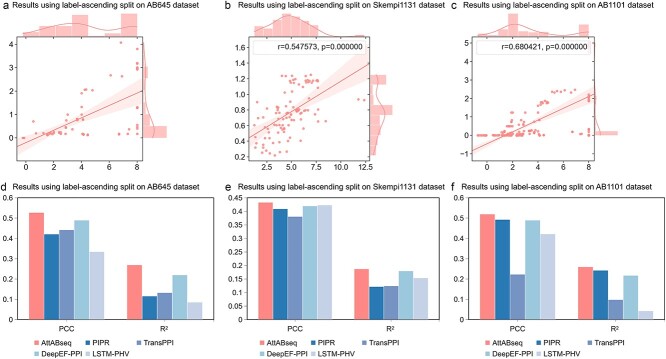
Evaluation on label-ascending ordered split on the three datasets; the evaluation metrics are PCC and R^2^. (a) Scatterplot of AttABseq on AB645 with label-ascending split; (b) Scatterplot of AttABseq on S1131 with label-ascending split; (c) Scatterplot of AttABseq on AB1101 with label-ascending split; (d) Performance comparison with four sequence-based methods on AB645 with label-ascending split; (e) Performance comparison with four sequence-based methods on S1131 with label-ascending split; (f) Performance comparison with four sequence-based methods on AB1101 with label-ascending split.

Given the scarcity of publicly available antigen–antibody complex data encompassing ∆∆G information pertaining to diverse mutations, an alternative approach is necessary to evaluate the efficacy of AttABseq. To enhance our model’s assessment, we organized the ∆∆G values in ascending order. Subsequently, we partitioned the antigen–antibody complex dataset into separate training and testing sets for AB645, AB1101, and S1131, adopting a label-ascending ordered split testing methodology. The results unequivocally demonstrate the superior performance of AttABseq compared with the other evaluated methods, as portrayed in Fig. 3. Notably, AttABseq achieved PCC values of 0.528 for AB645, 0.519 for AB1101, and 0.470 for S1131. This consistency across diverse dataset partitioning approaches underscores the generalization capability of AttABseq, confirming its significant practical utility in real-world scenarios.

### Comparison with structure–based approaches

Notably, FoldX [22] and BeAtMuSiC [23] are well-established structure–based tools renowned for their affinity change prediction capabilities and robustness. They have been recurrently employed as the benchmarks for method evaluation. In the context of the label-ascending split dataset experiment, our aim is to gage the precision and resilience of AttABseq in comparison to these alternative structure–based approaches.

Significantly, AttABseq emerges as the superior performer when contrasted with structure–based methods as shown in Table 1, which heavily rely on antigen–antibody structures. The constrained applicability of physics-based models lies in their primary focus on elucidating interaction mechanisms, rendering them less suitable for guiding antibody optimization routes and screenings [25]. Moreover, the inherent structural instability of the antibody’s hypervariable region within the major histocompatibility complex segment introduces a level of complexity that can challenge model predictions [65]. This underscores the need of leveraging DL techniques with protein sequences in the domain of antibody optimization and emphatically highlights the superior performance of AttABseq.

**Table 1:** The comparision results of AttABseq with 2 structure-based methods.

	AB645 dataset		S1131 dataset		AB1101 dataset	
Models	PCC	R2	PCC	R2	PCC	R2
AttABseq	**0.60**	**0.36**	**0.55**	**0.30**	**0.68**	**0.46**
FoldX	0.14	0.02	0.45	0.20	0.13	0.02
BeAtMuSiC	0.10	0.01	0.36	0.13	0.23	0.05

### Performance of AttABseq for therapeutic antibody optimization

Epidemic viruses pose a constant threat to global human life safety. To mitigate the harmful impact of viral infections on human health, researchers are employing antibody therapeutics for disease treatment. Here, we investigated the screening and optimization of specific antibodies for the two currently most devastating and widespread viruses: SARS-CoV-2 and Ebola. We systematically curated datasets comprising labels and data for two distinct classes of antibodies—one targeting the SARS-CoV-2 virus and the other targeting the Ebola virus—derived from an extensive review of the publicly accessible scientific literature. All the data and associated labels have been authenticated through rigorous biological experimentation. To demonstratively assess the efficacy of AttABseq in addressing challenges in antibody optimization, we implemented a ranking consistency evaluation in both external datasets. This was done to ascertain the alignment between the predicted outcomes of the model and the empirically derived labels, utilizing the R^2^ statistic as a measure of rank correlation.

**Figure 4:**
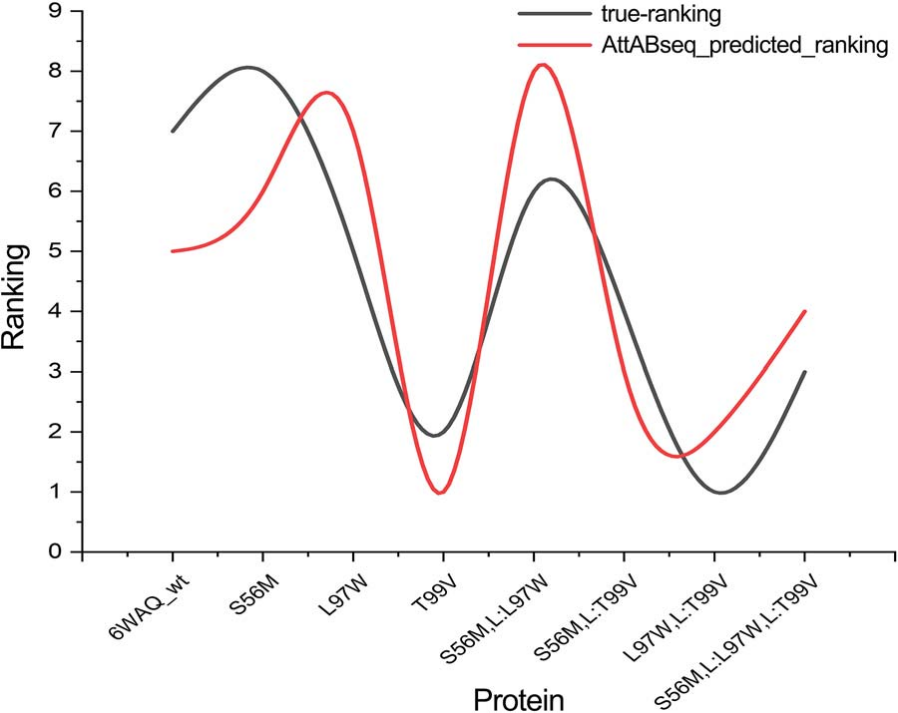
Performance of AttABseq for VHH optimization targeting the SARS-CoV-2 virus. The horizontal axis represents the antibody mutations, and the vertical axis denotes the ranking of antibody optimization outcomes. The line labeled “true-ranking” represents the ranking of antibody optimization outcomes arranged in ascending order based on their ∆k_D_ values, while the line labeled “AttABseq_predicted_ranking” indicates the rankings derived from ascending the ∆∆G values. AttABseq offers precise screening capabilities for the optimized selection of VHH antibodies that target the SARS-CoV-2 virus.

The emergence of the severe SARS-CoV-2, the causative agent of COVID-19, sparked a global pandemic in late 2019. To mitigate the virus’s spread and minimize its impact on human health, researchers have initiated a series of optimizations of specific antibodies targeting the SARS-CoV-2 spike protein, aiming to reduce the risk of mutational escape. Herein, referencing the work of Traian Sulea et al. [66], we conducted artificial intelligence–based antibody optimization experiments using the structure of the antigen–antibody complex 6WAQ. By introducing amino acid mutations, including S56M, L97W, and T99V, we simulated antibody optimization steps, employing the single-domain antibody (VHH) structure present in the complex. Subsequently, we calculated the ∆∆G values induced by amino acid mutations using AttABseq. We derived the change in dissociation constant (∆k_D_) by subtracting wild-type from mutant k_D_ values, which reflect changes due to amino acid point mutations, and ranked these from lowest to highest. A lower ∆k_D_ indicates superior antibody optimization. The AttABseq model’s predicted ∆∆G values were similarly ranked. This setup facilitated a ranking consistency evaluation between the observed and predicted data. The high consistency between the experimentally measured k_D_ values and the predictions, as shown in Fig. 4, highlights the reliability of the AttABseq model.

Given that the crystal structure data are derived from actual experiments, we have compared AttABseq with three popular structure-based antibody optimization tools: FoldX, BeAtMUSiC, and mCSM-AB2. As shown in Table 2, AttABseq not only surpasses the others in predicting the outcomes of VHH antibody optimization, accurately anticipating the effects of amino acid point mutations on antigen–antibody affinity changes, but it also pinpoints the most effective antibody optimization strategy. Notably, AttABseq relies less on structural data than other structure-based methods, easing the burden for experimental biologists in screening and optimizing antibodies through site-directed mutagenesis, thus offering greater practical value.

**Table 2:** Comparison with three structure-based methods for VHH optimization targeting the SARS-CoV-2 virus

Model name	R^2^
AttABseq	0.76
FolX	0.69
BeATMUSiC	0.16
mCSM-AB2	0.13

We applied AttABseq to recommend antibody optimization strategies for another target, the Ebola virus. The dataset was sourced from experiments [67]. However, the absence of published crystallographic data in the article precluded us from executing comparable experiments using structure-based antibody optimization tools. Nonetheless, AttABseq achieved an R^2^ value of 0.43 on this dataset. Further details and in silico experimental results are available at https://github.com/ruofanjin/AttABseq/External_test/Ebola.

### Significance of the attention block in AttABseq

**Figure 5:**
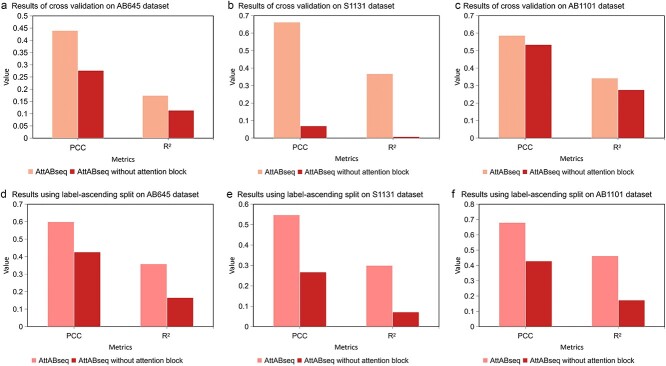
The importance of the attention block for AttABseq. The evaluation metrics are PCC and R^2^. From a to f, the results of the ablation study of the attention block have been shown.

To assess the pivotal role of our attention block in enhancing the model’s performance, we conducted an ablation study. The analysis primarily focuses on evaluating the AttABseq’s performance with and without the attention block. It is worth noting that the removal of the attention block does not affect the parameter size of the hidden layer. Moreover, we conducted the cross-validation and implemented a label-ascending ordered data split across the AB645, S1131, and AB1101 datasets, as depicted in Fig. 5. Under identical conditions for dataset partitioning, we performed training and testing on both AttABseq with and without the attention block. The results indicate the substantial enhancement in the AttABseq’s performance when the attention block is incorporated, compared to its performance without the attention block. This finding substantiates our assertion that the attention block significantly contributes to the understanding and effectiveness of AttABseq in addressing the complexities of antibody optimization arising from point mutations. This resource offers valuable insights and serves as a source of inspiration for future endeavors in the development of sequence-based biological models.

### Interpretability with the attention block of AttABseq

**Figure 6:**
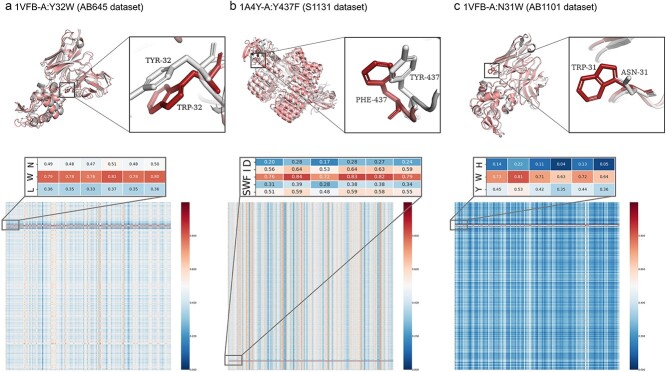
Visualization of antigen–antibody structure for interpretability study; the *x*-axis of the heatmap corresponds to the amino acid sequence of the antigen protein, while the *y*-axis represents the amino acid sequence of its corresponding antibody protein; (a) the MD simulations results of the 1VFB antigen–antibody complex with the mutation A:Y32W aligned with its wild-type; (b) the MD simulations results of the 1A4Y antigen–antibody complex with the mutation A:Y437F aligned with its wild-type, and the alteration in affinity may be attributed to the presence of the neighboring amino acid isoleucine (I) at the mutation site, and this could be due to the loss of the hydroxyl group resulting from the Y437F mutation, consequently disrupting the interaction between the 437th residue in the antibody and the fifth residue arginine (R) of the B chain; consequently, a novel contact force arises between the hydroxyl group of isoleucine and the fifth residue arginine (R) of the B chain, thereby exerting a more pronounced influence on the alteration of affinity, and (c) the MD simulations results of the 1VFB antigen–antibody complex with the mutation A:N31W aligned with its wild-type; the weight information for all three structures depicted as the heatmaps.

A notable strength of AttABseq is its capacity to offer granular insights at the residue level, enabling in-depth understanding of antibody optimization by visualizing the impact of residue mutations on the final predictive outcome using the attention block. Our analysis involved scrutinizing the weight of sequences derived from the attention block of the testing data, with a specific focus on residue mutations. The outcomes of this visualization are presented in Fig. 6, alongside a depiction of the crystal structure of an antigen–antibody complex obtained from the PDB file. Due to the limited availability of structural data, we performed molecular dynamics (MD) simulations to explore the interactions within the mutant variants. As a result, we obtained the alignment data for both the wild-type and mutated complexes. The weight information extracted from the attention feature map of the complex is subtracted from the attention block.

Effectively, the attention block harnesses weight information to illustrate the significance of the sequence data from both the antigen and antibody, enabling precise affinity predictions at the residue level. Our analysis affirms the conventional expectation that altered residues play a pivotal role in influencing changes in affinity. This information aligns seamlessly with the physicochemical attributes of sequence data, thereby demonstrating the model’s robustness and reliability in predicting affinity alterations resulting from point mutations.

## Discussion

### AttABseq’s performance in sequence-based antibody optimization

In this study, we proposed AttABseq as an innovative approach to in silico antibody optimization. Distinguishing itself from other ML methods for antibody optimization, AttABseq stands out as an end-to-end DL model by incorporating attention mechanisms and relying solely on protein sequences as its input. This unique approach positions AttABseq as a noteworthy advancement in the field. Through extensive cross-validation on benchmark datasets containing single and multi-mutations across diverse antigen–antibody complexes, AttABseq consistently outperforms traditional sequence-based approaches and even surpasses structure–based methods. These results highlight the computationally discernible link between amino acid point mutations and the affinity maturation process, reaffirming AttABseq’s efficacy.

### AttABseq’s advantage over structure-based antibody optimization methods

Traditional structure-based approaches often rely on the structural analysis of antigen–antibody complexes to estimate the thermodynamic free energy change (∆∆G) caused by mutations. However, the affinity maturation process predominantly occurs in the CDRs, characterized by their structural flexibility. Mutated protein structures, where only residue types change without the corresponding spatial adjustments among protein residues, often lack accuracy. This limitation may lead to ineffective or potentially detrimental predictions. In contrast, AttABseq excels by its capacity to provide an efficient and time-saving solution. It sidesteps the need for labor-intensive structural modeling, especially for mutated proteins, and relies solely on sequence information. As an end-to-end model, AttABseq emerges as the preferred choice for antibody optimization.

### Limitations and future work

Despite its significance in bridging computational antibody optimization and limited computing power, AttABseq faces constraints associated with the qualities and quantities of available data. The scarcity and considerable variations in the number of antigen–antibody complexes within datasets like AB-Bind and Skempi pose limitations. Additionally, the non-uniform sequence lengths across different antibody types present challenges. Expanding the current dataset for various antigen types, encompassing more antibody optimization solutions and databases, becomes imperative.

In the external validation trials, AttABseq reaffirmed its prowess, outstripping the predictive acumen of structure-based antibody optimization methodologies. Nonetheless, it has come to our attention that AttABseq’s predictive proficiency occasionally falters, especially in scenarios involving antibody optimization against antigens with particularly lengthy amino acid sequences. This reveals avenues for refinement and underscores the potential for enhancing the algorithm’s robustness in future iterations. Furthermore, with the increasing wealth of bio-related data in databases, the application of our model in protein engineering studies in biology and biomedicine holds significant promise. Particularly, its deployment on larger, experimentally validated datasets can shed light on the impact of amino acid species changes on protein properties. This is poised to offer improved solutions for protein drug design and screening, contributing to the broader field of bioengineering.

## Methods

### Datasets

In the pursuit of rigorous computational affinity predictions for antigen–antibody complexes, we leveraged two well-established and extensively utilized databases: AB-Bind [62] and Skempi 2.0 [63]. These repositories are renowned for their comprehensive collections of antigen–antibody complexes and have served as valuable resources in numerous computational studies [68, 69].

**Figure 7:**
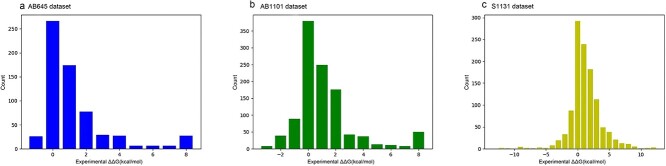
Data distribution of the AB-Bind dataset; (a) AB645 dataset consists of 29 types of antibodies with 645 mutated variations; (b) AB1101 dataset consists of 32 types of antibodies with 1101 mutated variations; (c) Skempi 2.0 dataset (S1131) consist of 112 types of antibodies with 1131 mutated variations.

AB-Bind, a repository of diverse antigen–antibody complexes, serves as a pivotal dataset for assessing methods that aim to predict the interactions of antibodies. It encompasses a rich array of 32 antigen–antibody protein complexes, each characterized by non-repetitive residue mutations, with binding affinity changes measured through meticulous wet experiments. Specifically, we extracted a subset from AB-Bind, referred to as AB645, in alignment with the methodology outlined by Pires et al. [24]. AB645 focuses exclusively on 645 single mutations distributed across 29 antibody–antigen complexes, thus forming a robust benchmark dataset. Please refer to Fig. 7a and b for a visual representation of the datasets AB645 and AB1101, both derived from the AB-Bind repository.

Skempi 2.0, another esteemed database, offers a meticulously curated compendium of binding data encompassing a broad spectrum of mutations across various protein complexes. Within this repository, we isolated a dataset known as S1131, comprised of 1131 mutations spanning 112 antigen–antibody protein complexes. These mutations have been employed in prior computational experiments aimed at predicting affinity changes [25, 27]. Figure 7c provides an illustrative overview of the dataset S1131, derived from the Skempi 2.0 repository. These carefully selected benchmark datasets serve as the foundation for our computational affinity predictions, offering a robust framework for the evaluation and advancement of our AttABseq model.

### Sequence feature descriptors

To represent protein sequences as matrices, we primarily employ two feature types, one-hot matrix (OHM) and PSSM.

OHM is a sequence featurization method to encode sequences, specifically amino acid sequences or linear arrangements of other data types. It converts each category in the input sequence into a one-hot vector, where only one element is set to 1, indicating the presence of that category, while the rest are set to 0. The position of the ‘1’ element corresponds to the index of the category. For example, assuming that a categorical variable with *n* categories (indexed from 1 to *n*) has a value of *i* (1 ≤ *i* ≤ *n*), the corresponding formula for the one-hot encoding matrix *X* is:


$$ {X}_{ij}=\left\{\begin{array}{c}1\kern0.5em \mathrm{if}\ i=j\\{}0\kern0.5em \mathrm{if}\ i\ne j\end{array}\right..$$

where ${X}_{ij}$ is the element in the *i*th row and *j*th column of the one-hot encoding matrix. ${X}_{ij}$ will be set to 1 when $i=j$ and ${X}_{ij}$ will be set to 0 when $i\ne j$.

PSSM is a highly regarded data structure renowned for its exceptional ability to capture position-specific details within biological sequences, particularly in proteins and deoxyribonucleic acid (DNA). This matrix-based encoding has become a pivotal computational tool in representing protein sequences [70, 71]. Numerous DL studies focused on protein sequences have leveraged the PSSM’s capability to unveil complex sequence features [72–75]. Here, the PSSM is generated using PSI-BLAST [76] and sources from the UniProtKB/Swiss-Prot database. Through PSSM featurization, we can derive a PSSM of size $\left(n,20\right)$ from a protein sequence containing $n$ amino acids. Detailed parameters and commands used for PSSM generation are available in our GitHub repository (https://github.com/ruofanjin/AttABseq).

Therefore, we can generate an OHM with a shape of $\left(n,20\right)$ and a PSSM with a shape of $\left(n,20\right)$ from a protein sequence consisting of $n$ amino acids. Subsequently, the concatenation of the OHM and the PSSM results in a comprehensive input feature matrix with a shape of $\left(n,40\right)$ that represents the protein sequence in our study.

### Model design

In this study, we introduce AttABseq, which utilizes full-length protein sequence data from both wild-type and mutant antigen–antibody complexes for model training. Since AttABseq is used to predict the affinity change values resulting from amino acid point mutations in antigen–antibody complexes, we categorize the input data into four types: the antibody sequence of the wild-type complex, the antigen sequence of the wild-type complex, the antibody sequence of the mutant complex, and the antigen sequence of the mutant complex. We then extract sequence feature descriptors from these four sets of data as the model input features: ${Feature}_{ab}^{wt}$, ${Feature}_{ag}^{wt}$, ${Feature}_{ab}^{mt}$, and ${Feature}_{ag}^{mt}$. These features are used to represent the four different types of sequence data.

**Figure 8:**
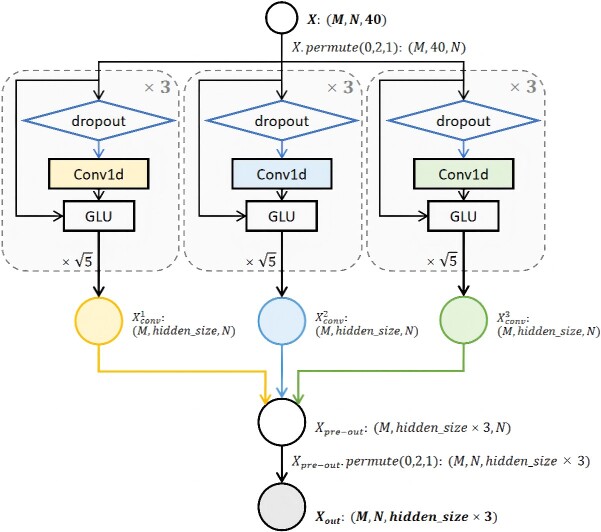
The embedding block architecture of AttABseq, and the shape of the input features is $\left(M,N,40\right)$, where $M$ represents the batch size set for AttABseq, and after passing through the embedding block, the final model yields an ${X}_{out}$ of size $\left(M,N, hidden\_ size\times 3\right)$.

In the construction of our AttABseq, once the sequence features have been effectively extracted, the subsequent stage involves the embedding block, which aims to delve deeper into the nuances of protein characteristics. In the embedding block, ${Feature}_{ab}^{wt}$, ${Feature}_{ag}^{wt}$, ${Feature}_{ab}^{mt}$, and ${Feature}_{ag}^{mt}$ are processed using a series of convolutional networks employing Conv1D [77] and gated linear units. We applied the same embedding step to all four types of features. Here, we define the input to the embedding block as $X$ and its output as ${X}_{out}$ in Fig. 8 and the hidden size is represented as $hidden\_ size$ to illustrate the details regarding the embedding block. The combined vector ${X}_{out}$ is subsequently passed through a Layer Normalization module for vectors normalization. Here, we use $torch. nn. LayerNorm$. More detailed information about the parameters used for training such as hidden size and the kernal size of the module $Conv1d$ are available in our GitHub repository (https://github.com/ruofanjin/AttABseq). Based on the inputs ${Feature}_{ab}^{wt}$, ${Feature}_{ag}^{wt}$, ${Feature}_{ab}^{mt}$, and ${Feature}_{ag}^{mt}$, we obtain ${Embedding}_{ab}^{wt}$, ${Embedding}_{ag}^{wt}$, ${Embedding}_{ab}^{mt}$, and ${Embedding}_{ag}^{mt}$ after the embedding block.

Following the embedding block, the attention block, a pivotal component of our model, incorporates a multi-head self-attention module designed to enhance the individual protein features. Concurrently, it firstly employs a multi-head mutual-attention module to establish intricate associations between antigens and antibodies within each complex. This multi-head mutual-attention module facilitates the seamless transfer of information through feature vectors, taking into account their respective mapping relationships. The computational steps are detailed in Equations 1 and 2


(1) \begin{equation*} {M}_{self- attention}= Dropout\left( softmax\left(\frac{W_qM\cdotp{W}_k{M}^T}{\sqrt{d_k}}\right)\cdotp{W}_vM\right)+M \end{equation*}

(2) \begin{align*} &{Complex_i}_{mutual- attention}\nonumber\\&= softmax\left(\frac{W_q{Complex}_i\cdotp{W}_k{Complex_j}^T}{\sqrt{d_k}}\right)\cdotp{W}_v{Complex}_j. \end{align*}

In Equation 1, $M$ represents the input and ${M}_{self- attention}$ signifies the output. The $W$ is weight with subscript $q$, $k$ and $v$ correspond to queries, keys and values, respectively. $\sqrt{d_k}$ is a scaling factor depending on the layer size number. In the attention block, through Equation 1, we calculate ${M_{self- attention}}_{ab}^{wt}$, ${M_{self- attention}}_{ag}^{wt}$, ${M_{self- attention}}_{ab}^{mt}$, and ${M_{self- attention}}_{ag}^{mt}$ from ${Embedding}_{ab}^{wt}$, ${Embedding}_{ag}^{wt}$, ${Embedding}_{ab}^{mt}$, and ${Embedding}_{ag}^{mt}$, respectively, obtained from the embedding block. Subsequently, based on whether the protein source originates from wild-type or mutant, we categorize the features into two groups, namely ${Complex}_{wt}$ and ${Complex}_{mt}$. In Equation 2, ${Complex}_i$ and ${Complex}_j$, respectively, represent the two types of proteins within the same protein complex group $Complex$. Based on Equation 2, we perform the same type of feature calculation for the two sets of data, ${Complex}_{wt}$ and ${Complex}_{mt}$, and then calculate ${Complex}_{i_{mutual}}^{wt}$, ${Complex}_{j_{mutual}}^{wt}$, ${Complex}_{i_{mutual}}^{mt}$, and ${Complex}_{j_{mutual}}^{mt}$. Also, the 𝑊 is the weight and its subscripts *q*, *k*, *v* are associated with queries, keys, and values, while $\sqrt{d_k}$ is a scaling factor contingent to the layer size number. Through the integration of Equations 1 and 2, the multi-head mutual-attention module adeptly captures the protein sequence information inherent in wild-type and mutant antigen–antibody complexes, thereby encapsulating a nuanced characterization of complex-specific information.

**Figure 9:**
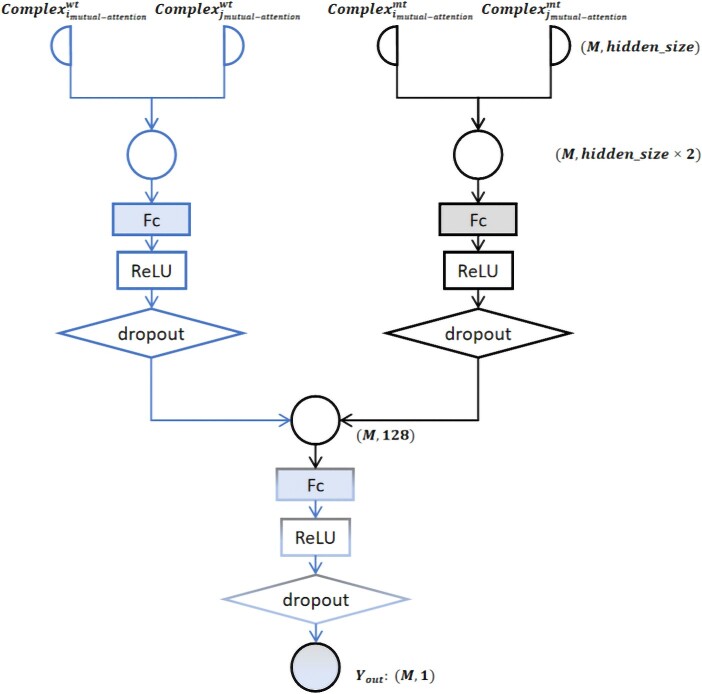
The prediction block architecture of AttABseq.

The final component of our model, the prediction block, is tasked with generating the ultimate predictions for ΔΔG in wild-mutant antigen–antibody complex pairs. First, we concatenating the representations of wild-type and mutated-type antigen–antibody complexes into two vectors. These combined vectors undergo processing through three fully connected layers of varying sizes. This final step culminates in the regression prediction of antibody affinity changes as shown in Fig. 9.

During model training, we calculate mean squared error (MSE) to describe the difference between model-predicted values and true values, and we use torch.nn.MSELoss as our loss function. For example, we have two lists of $X$ and $Y$ with the same length, and their MSE loss is calcuted as


$$ MSE=\frac{1}{N}\sum_{i=1}^n{\left({x}_i-{y}_i\right)}^2, $$

where ${x}_i$ is the $i$th value in $X$ and ${y}_i$ is the $i$th value in $Y$. $N$ is the length of the list $X$ or $Y$, $n$ is the biggest index in the list $X$ or $Y$.

### Model evaluations

In this study, we prioritize the prediction of affinity changes in antigen–antibody interactions caused by missense mutations as a pivotal aspect of computational antibody optimization. To this end, we have devised the AttABseq model, which directly infers numerical affinity alterations. Consequently, in our experimental designs, we employ two distinct evaluation metrics: the PCC and R^2^, each offering a unique perspective on model performance. PCC, also known as Pearson correlation, is used to measure the strength and direction of the linear relationship between two continuous variables. R^2^ is the proportion of the squared differences between actual observations and model predictions, relative to the proportion of squared differences between actual observations and their mean.

### Experiments

In light of the relatively limited scale of our datasets, we devised two distinct experimental approaches.

The first approach hinges on K-fold cross-validation, a widely adopted technique in ML experiments. K-fold cross-validation involves repeatedly splitting the dataset into different subsets, enabling us to derive robust and unbiased performance metrics by averaging results across multiple iterations. This strategy serves to mitigate the potential challenges posed by non-uniform data distribution within a single partition. Cross-validation is a fundamental empirical practice for assessing the generalizability of ML algorithms across various datasets. In our experiments, we employed five-fold validation for the AB1101 dataset and ten-fold validation for both the AB645 and S1131 datasets. Each fold is created through a randomized process to ensure impartiality.

The second approach is the label-ascending dataset split method for the experiment to figure out the robustness of our model. This approach offers consistent partitioning protocols across different datasets, reducing the need for extensive training and evaluation times while simultaneously assessing the model’s capacity to handle diverse data intricacies. With this method, data are initially organized based on the antigen–antibody taxonomy. Subsequently, mutant labels for each complex are arranged in ascending order. By adhering to predefined ratios, we select the subset containing the highest labels within the mutant group for each complex as our test set. The remaining subset, comprising a smaller proportion of data, is designated as the training set. After this division, our model and baselines are trained and evaluated using these distinct datasets. This strategy ensures a comprehensive evaluation of our model’s performance across different data distributions and complexities.

### A uniform feature transformation approach for interpretability

Here, we proposed a uniform feature transformation approach to interpret the biochemical mechanism of antigen–antibody affinity change due to single-point and multi-points amino acid mutation.

Since the weight was extracted from the attention block, we now have gotten the weight of wild-type antigen–antibody interaction (${W}_{Ag-{Ab}^{wt}}$) and the weight of mutated-type antigen–antibody interaction (${W}_{Ag-{Ab}^{mt}}$). At this point, the weight matrix for the antigen–antibody has the shape of (${length}_{Ag},{length}_{Ab}$). Consequently, these weights can be conceptualized as a 2D sequence-based antigen–antibody interaction map. Each grid point can be seen as any amino acid pair on the antigen–antibody sequences, and the numerical value at each grid point represents the digitized characterization of the interaction between those amino acid pairs.

To facilitate a meaningful comparison between the wild-type antigen–antibody and mutant antigen–antibody, we first normalized their weight matrices. This normalization process ensures that each value in the matrix falls within a standardized range, allowing for a fair comparison


(3) \begin{equation*} \mathit{\operatorname{norm}}(x)=\frac{x-\mathit{\min}(x)}{\mathit{\max}(x)-\mathit{\min}(x)} \end{equation*}

(4) \begin{equation*} {Wnew}_{Ag-{Ab}^{wt}}=\mathit{\operatorname{norm}}\left({W}_{Ag-{Ab}^{wt}}\right) \end{equation*}

(5) \begin{equation*} {Wnew}_{Ag-{Ab}^{mt}}=\mathit{\operatorname{norm}}\left({W}_{Ag-{Ab}^{mt}}\right). \end{equation*}

Subsequently, we subtracted the normalized weight matrix of the mutant antigen–antibody, ${Wnew}_{Ag-{Ab}^{mt}}$, from that of the wild-type antigen–antibody, ${Wnew}_{Ag-{Ab}^{wt}}$, resulting in a new weight matrix, ${W}_{distance}$. After normalizing the new weight matrix, we obtained a matrix, ${Wnew}_{distance}$, which can be used to represent the impact of affinity changes between any pair of amino acids in the antigen–antibody sequences before and after mutations


(6) \begin{equation*} {W}_{distance}={Wnew}_{Ag-{Ab}^{mt}}-{Wnew}_{Ag-{Ab}^{wt}} \end{equation*}

(7) \begin{equation*} {Wnew}_{distance}=\mathit{\operatorname{norm}}\left({W}_{distance}\right). \end{equation*}

**Key Points**

- A deep learning model called AttABseq that can predict the antigen–antibody binding affinity changes based on sequence information was developed.
- AttABseq exhibited the highest prediction accuracy and superior generalization compared with other sequence-based models.
- The molecular mechanisms behind antigen–antibody binding affinity changes induced by point mutations can be revealed by the attention block of AttABseq.

## Data Availability

The data can be found at https://github.com/ruofanjin/AttABseq.

## Author Biographies

**Ruofan Jin** is currently a PhD student in the College of Life Sciences at Zhejiang University under the supervision of Prof. Tingjun Hou, Prof. Ruhong Zhou, and Prof. Chang-Yu Hsieh. Her research interests mainly lie in the area of computer-aided drug design.

**Jike Wang** is currently a postdoctoral fellow in the College of Pharmaceutical Sciences at Zhejiang University. His research interests mainly lie in the area of computer-aided drug design.

**Zheng Cao** is currently a PhD student under the supervision of Prof. Jian Wu in the College of Computer Science and Technology at Zhejiang University. His research interests include artificial intelligence for science and computer-aided diagnosis.

**Dejun Jiang** is currently a postdoctoral fellow at the Innovation Institute for Artificial Intelligence in Medicine of Zhejiang University in the College of Pharmaceutical Sciences at Zhejiang University. His research interests mainly lie in the area of computer-aided drug design.

**Qing Ye and Tianyue Wang** are currently PhD students in the College of Pharmaceutical Sciences at Zhejiang University under the supervision of Prof. Tingjun Hou. Their research interests mainly lie in the area of computer-aided drug design.

**Yu Kang** is currently an associate professor in the College of Pharmaceutical Sciences at Zhejiang University. Her research interests are mainly in the area of computer-aided drug design and molecular modeling.

**Wanting Xu** is currently a master student in the College of Pharmaceutical Sciences at Zhejiang University under the supervision of Prof. Tingjun Hou. Her research interests mainly lie in the area of computer-aided drug design.

**Chang-Yu Hsieh** is currently a professor in the College of Pharmaceutical Sciences at Zhejiang University. His research interests are mainly in the area of artificial intelligence for science and computer-aided drug design.

**Tingjun Hou** is currently a professor in the College of Pharmaceutical Sciences at Zhejiang University. His research interests can be found at the website of his group: http://cadd.zju.edu.cn.
